# Phospholipids and phospholipase A_1_ as antigens during the course of experimental *Trypanosoma cruzi* infection

**DOI:** 10.1590/0074-02760240281

**Published:** 2025-09-15

**Authors:** Emanuel Bott, Sebastián Andrés López, Guadalupe Gimenez, María Elisa Solana, María Laura Belaunzarán

**Affiliations:** 1 Universidad de Buenos Aires, Facultad de Medicina, Departamento de Microbiología, Parasitología e Inmunología, Buenos Aires, Argentina; 2 Consejo Nacional de Investigaciones Científicas y Técnicas-CONICET, Universidad de Buenos Aires, Instituto de Investigaciones en Microbiología y Parasitología Médica, Buenos Aires, Argentina; 3 Universidad Nacional de Luján, Departamento de Ciencias Básicas, Buenos Aires, Argentina

**Keywords:** antibodies, antiphospholipid, phospholipases A_1_, phospholipids, Trypanosoma cruzi

## Abstract

**BACKGROUND:**

*Trypanosoma cruzi,* causative agent of Chagas disease (CD), remains a public health problem in Latin America and is emerging in non-endemic areas. Phospholipids (PL) are essential components of biomembranes and their enzymatic modification by phospholipases yields bioactive lipids that modulate immune responses. Anti-PL antibodies have been associated with autoimmune diseases and inflammation, potentially influencing CD pathology by recognising PL and PL-binding proteins. *T. cruzi* Phospholipase A_1_ (TcPLA_1_) hydrolyses membrane PL and participates in parasite-host cell interactions.

**OBJECTIVES:**

This study evaluated IgM and IgG antibody responses against phosphatidylcholine, phosphatidylethanolamine, and their derived lysophospholipids (LPL), as well as recombinant TcPLA_1_, during experimental *T. cruzi* infection with two strains: RA (high virulence) and K98 (low virulence). It also aimed to predict the recognition capacity of TcPLA_1_ by CD patients using *in silico* analysis.

**METHODS:**

Antibody responses were analysed by enzyme-linked immunosorbent assay (ELISA) using different PL and recombinant TcPLA_1_ as antigens. Lytic activity assays were performed to evaluate the functional impact of anti-PL antibodies. The CHAGASTOPE resource was used to predict TcPLA_1_ antigenicity.

**FINDINGS:**

This study identified IgM and IgG antibodies against PL, LPL and TcPLA_1_ during experimental *T. cruzi* infection. Different amino acid sequences of TcPLA_1_ showed stronger antigenic recognition by CD patient’s sera.

**MAIN CONCLUSIONS:**

The presence of these antibodies suggests their involvement in the pathogenesis of CD and their potential as markers for disease monitoring and prognosis.

*Trypanosoma cruzi*, the etiological agent of Chagas disease (CD), represents a public health problem in Latin America and has spread to non-endemic areas in recent decades due to human migration. According to the World Health Organisation, approximately more than 7 million people worldwide are estimated to be infected with this protozoan parasite.^1^ *T. cruzi* is highly heterogeneous in terms of genetics and biological behaviour, and great efforts have been made to identify molecules involved in parasite-host cell interaction that may also contribute to the pathogenesis of CD.^2^

Phospholipids (PL), major components of biomembranes, can be enzymatically modified by the action of phospholipases with generation of bioactive lipids that can act as second messengers and also modulate the immune response.^3^ In animal cells, the most abundant PL are phosphatidylethanolamine (PE) and phosphatidylcholine (PC), which contain respectively ethanolamine and choline as polar heads, with other important PL being phosphatidylserine and phosphatidylinositol.

Anti-PL antibodies are immunoglobulins of the IgG, IgM or IgA isotype that recognise PL and/or plasma proteins linked to PL. The latter found mostly in autoimmune pathologies are capable of creating a neo-epitope by changing PL-bound-protein conformation or unmasking antigenic cryptic protein epitopes.^4^ Regarding the structural features that can influence the binding affinity and specificity of anti-PL antibodies, it has been described for anti-PC antibodies that the length and/or degree of saturation of fatty acid chains, as well as the polar head group, may serve as relevant epitopes for their recognition.^4^ ^-^ ^10^

The induction of anti-PL antibodies and the development of antiphospholipid syndrome have been associated with viral, bacterial, and protozoan infections, with cardiolipin, an anionic PL, being a strong marker of this pathology.^11^ The presence of anti-PL antibodies has been described in diseases related to tissue inflammation, immunological disorders, pregnancy and aging,^12^ as well as in those caused by protozoan parasites such as *Babesia bovis*, *Plasmodium falciparum* and Trypanosomatids.^13^ ^,^ ^14^ ^,^ ^15^ ^,^ ^16^ In CD chronic patients, anti-*T. cruzi* and anti-cardiolipin antibodies of the IgM and IgG isotype have been detected. In this sense, cardiolipin could represent an antigenic stimulus that contributes to CD cardiomyopathy due to its vast distribution in heart tissue.^15^

The first evidence for PL-degrading enzymes in *T. cruzi* infection was associated with the inflammatory responses observed around degenerating amastigote nests in various tissues of CD patients, suggesting that inflammation could be partially caused by molecules derived from PL hydrolysis, such as free fatty acids (FFA) and lysophospholipids (LPL).^17^ ^,^ ^18^ In line with this finding, our research has shown that the extensive degradation of endogenous PL with accumulation of FFA and LPL was attributed to the activity of *T. cruzi* Phospholipase A_1_ (TcPLA_1_).^19^ ^,^ ^20^ Besides, TcPLA_1_ has been considered as a virulence factor since this membrane bound/secreted enzyme is able to generate second lipid messengers that activate protein kinase C, required for parasite invasion.^21^ According to this fact, antibodies raised against TcPLA_1_ significantly reduced *in vitro* parasite invasion, indicating that this enzyme is involved in early events of parasite-host cell interaction.^22^

In the present study, we investigated the IgM and IgG antibodies response against PC, PE, LPC, LPE and TcPLA_1_ during murine *T. cruzi* experimental infection, with two different parasite strains: RA (high virulence) and K98 (low virulence) and evaluated the potential lytic activity of anti-PL antibodies. Besides, we performed immunoinformatics to evaluate TcPLA_1_ antigenicity in CD patients.

## MATERIALS AND METHODS

*Materials* - MaxiSorp plastic microplates were obtained from Nunc (Roskilde, Denmark). HisPur Ni-NTA Spin Column, Penicillin-Streptomycin solutions, RPMI 1640 culture media and Tetramethylbenzidine (TMB) substrates were from Thermo Fisher Scientific Inc. (Waltham, MA, USA). The biotechnological quality foetal bovine serum (FBS) was acquired from Internegocios S.A. (Mercedes, Buenos Aires, Argentina). Protease inhibitor cocktail, Nα-Tosyl-Lys-chloromethylketone (TLCK), trans-epoxysuccinyl-L-leucylamide-(4-guanidino)-butane (E-64), sodium azide, tris-(hydroxymethyl)-aminoethane (TRIS), glycine, Tween- 20, bovine serum albumin (BSA) fraction V, lipid standards, were obtained from Sigma Chemical Co. (St. Louis, MO, USA). Analytical grade organic solvents were purchased from Merck Biosciences (Darmstadt, Germany). Bio-Rad protein determination reagent was obtained from Bio-Rad Laboratories (Hercules, CA, USA). Peroxidase-conjugated anti-mouse IgG, anti-goat IgG, and anti-rabbit IgG antibodies were obtained from Santa Cruz Biotechnology Inc (CA, USA).

*Ethics statement* - To carry out this work, BALB/c mice were bred and maintained at the animal facilities of the Instituto de Investigaciones en Microbiología y Parasitología Médica (IMPaM, Consejo Nacional de Investigaciones Científicas y Técnicas - Universidad de Buenos Aires), Buenos Aires, Argentina. All animal procedures were approved by the Institutional Committee for the Care and Use of Laboratory Animals (CICUAL, Facultad de Medicina, Universidad de Buenos Aires), in line with guidelines provided by the Administración Nacional de Medicamentos, Alimentos y Tecnología Médica (ANMAT), Servicio Nacional de Sanidad y Calidad Agroalimentaria (SENASA) from Argentina and based on the US NIH Guide for the Care and Use of Laboratory Animals.

*Parasites* - Two different *T. cruzi* strains, each belonging to a different discrete typing unit (DTU), were used in this study: RA (TcVI, high virulence) and K98 (TcI, low virulence).^23^ ^,^ ^24^

Epimastigote cultures were performed with parasites from the RA strain, that were axenically grown in a biphasic medium at 28ºC, as previously described.^25^

Bloodstream trypomastigotes of the RA strain were maintained by weekly passages in 21-day-old CF1 mice and bloodstream trypomastigotes of the K98 strain were maintained by passages every 45 days in 21-day-old CF1 mice^23^ at the animal facilities of the IMPaM. For further serum collection, bloodstream trypomastigotes from both strains were collected separately at the peak of parasitaemia, counted on a Neubauer chamber, diluted in phosphate-buffered saline (PBS)-1% bovine serum albumin (BSA), and used to infect via intradermal injection two groups of two-month-old male BALB/c mice (n = 8 per group) with 1500 bloodstream trypomastigotes from RA or K98 strains in the hind paw pad.

*Serum collection* - Blood samples were collected from each group of mice at three time points: before the experimental infection (day 0), during the acute phase [21, 30, 42, and/or 54 days post-infection(dpi)], and during the chronic phase of infection (120 dpi). The choice of classification as acute (corresponding to the peak of parasitaemia) or chronic (after the peak of parasitaemia), to allow a biologically meaningful comparison, is related to the different duplication times of each strain, which cause the peaks of parasitaemia to be reached at different times - RA peaks at 9 dpi, whereas K98 peaks at 45 dpi. Blood samples were left to clot for at least 30 minutes at room temperature (RT) and then centrifuged at 10,000×g for 5 min at RT to separate serum. Samples were then aliquoted and stored at -20ºC for until used in enzyme-linked immunosorbent assay (ELISA) assays.

*Detection of anti-PL antibodies in T. cruzi infected mice by ELISA* - This protocol was carried out as previously described by Gimenez et al.^26^ MaxiSorp plates were sensitised with 100 μL of each commercial lipid (LPC, LPE, PC and PE) dissolved in ethanol (50 μg/mL). After ethanol was evaporated at 37ºC, plate blocking was performed with 200 μL of 2% BSA solution in Tris buffered saline (TBS), for 2 h at 37ºC. Plates were then washed five times with TBS and incubated with 50 μL of sera from mice infected with RA or K98 *T. cruzi* strains, diluted 1:100 (v/v) in 1% BSA solution in TBS, for 2 h at 37ºC. Plates were washed five times with TBS, incubated with 50 μL of the conjugates anti-murine IgM-HRP 1:500 (v/v), anti-murine IgG-HRP 1:5000 (v/v) for 2 h at 37ºC and after being washed with TBS five times, they were incubated for 10 min with 100 μL of TMB. The reaction was stopped with 50 μL 4N H_2_SO_4_ and the absorbance was determined at 450 nm (A450) in a microplate reader (RT-6000, Bio Rad). The A450 measurement on day 0 represents the antibody levels in uninfected mice. This baseline measurement serves as a reference point to compare the increase in antibody levels in infected mice compared to uninfected mice.

*Determination of the lytic activity of anti-PL antibodies in sera of T. cruzi infected mice* - This method was adapted from the protocol described by Gimenez et al.^26^ In order to test lytic activity of anti-PL antibodies present in *T. cruzi* infected mice, sera pre-adsorbed with PC/PE liposomes or mocked sera were incubated with viable epimastigotes and their number was registered.

*Liposomes preparation* - Liposomes were prepared by mixing 5 mg of PC + 5 mg of PE in chloroform for a final volume of 1 mL. The organic solvent was evaporated under the N_2_ atmosphere and the lipids were suspended and hydrated in a solution of 9% W/V Ficoll-paque and 60% (v/v) meglumine in PBS, for 30 min at RT. Then, they were vigorously mixed by vortexing in the presence of glass spheres and then sonicated for 5 min with a Torbeo Sonicator (Cole Palmer).

*Adsorption of murine sera with liposomes* - The PC/PE liposome suspension (70 μL) was added to 180 μL pooled sera from mice infected with the K98 strain in the chronic phase (120 dpi). The mixture was incubated for 1 h at RT with gentle shaking, centrifuged (15,000 g, 20 min) and the supernatant collected to test its lytic activity (sera pre-adsorbed with liposomes). This pooled serum was previously inactivated for 30 min at 56ºC, prior to use in the lytic activity assays, in order to inactivate the source of endogenous complement.

*Lytic activity assay* - A suspension of 250 μI of *T. cruzi* epimastigotes, RA strain, (5 x 10^5^ total parasites), diluted in 1% BSA-PBS was incubated with 250 μI of 1/8 dilution of: (i) inactivated sera from *T. cruzi*-infected mice (Tc) with or (ii) inactivated sera from non-infected mice (N) or (iii) or inactivated sera from *T. cruzi* infected mice that were pre-adsorbed with PC/PE liposomes to remove anti-PL antibodies (-αPL). Complement activity of sera samples was evaluated by determining the dilution of sera required to lyse approximately 50% of parasites (limiting dilution assay).^27^ The assays were performed in the presence or absence of an external source of complement (C´) (10 μI of non-immune guinea-pig serum), for 30 min at 28ºC. Aliquots were then taken, mixed with an equal volume of 4% paraformaldehyde to fix the parasites and then counted in a Neubauer chamber by microscopy.

*Expression of recombinant TcPLA*_*1*_ - TcPLA_1_ (GenBank Accession number: JN975637.1) was expressed in the baculovirus expression vector system (BEVS), using Bac-to-Bac™ methodology (Thermo Fisher Scientific Inc.) as previously described.^20^

For TcPLA_1_ purification, Sf9 baculovirus infected cells (10^7^) were washed twice with PBS, suspended in 1 mL of Lysis buffer (50 mM NaH_2_PO_4_, 300 mM NaCl, 10 mM Imidazole, pH 7.6) in the presence of 1X protease cocktail inhibitor and incubated at 4ºC for 30 min. The lysate was centrifuged (10,000×g, 20 min, 4ºC) and the clear supernatant was collected and filtered through 0.45 μm membrane to be further loaded onto a HisPur Ni-NTA Spin Column for affinity purification of His-tagged TcPLA_1_. After washing with Lysis buffer (10v) recombinant TcPLA_1_ was eluted by pH change using 50 mM NaH_2_PO_4_, 300 mM NaCl, pH 4-5. After recombinant protein purification, aliquots were analysed by SDS-PAGE, followed by Coomassie blue staining and immunoblot assay using either an anti-histidine primary antibody or anti-*T. cruzi* PLA_1_ serum, as previously described.^20^

*Detection of anti-TcPLA*_*1*_ *antibodies in T. cruzi infected mice by ELISA* - MaxiSorp plates were sensitised with 50 μL of recombinant TcPLA_1_ in PBS (5 μg/mL) overnight at 4ºC. Plates were then washed three times with 0.05% Tween-20-PBS (PBST) and blocked with 200 μL of a 2% BSA solution in PBS for 1 h at 37ºC. The plates were washed five times with PBST and incubated for 1 h at 37ºC with 50 μL of each serum from mice infected with RA or K98 *T. cruzi* strains, obtained at different dpi, diluted in a 1% BSA solution in PBS (1:100 dilutions). The plates were washed five times with PBST, incubated with 50 μL of the conjugates anti-murine IgM-HRP 1:500 (v/v) or anti-murine IgG-HRP 1:5000 (v/v) diluted in BSA to 1 % in PBS, for 1 h at 37ºC and after washing five times with PBST, they were incubated for 10 min with 50 μL of TMB. The reaction was stopped with 50 μL of 4N H_2_SO_4_ and the absorbance was measured at 450 nm in a microplate reader (RT-6000, Bio Rad).

*Analyses of TcPLA*_*1*_ *antigenicity by immunoinformatics* - To identify antigenic regions of TcPLA_1_ (GenBank: AEX65839.1), the Chagas Antigen and Epitope Atlas resource CHAGASTOPE-v2 was used.^28^ Considering that the platform explores the antigenicity of predicted *T. cruzi* proteomes, we used for the antigen search the corresponding homologous sequences of TcPLA_1_: TcCLB.511439.20 and TcCLB.510681.30, belonging to the CLBrener31 reference strain, and searched for antibody-binding signals for peptides grouped by protein and by antigenic region. Results of these *in silico* analyses were displayed as graphs, which were constructed by the comparison of fluorescence signals for each peptide position of TcPLA_1_ obtained with a pool of sera from patients infected with *T. cruzi* and another pool of uninfected controls (positive and negative pools, respectively).

*Statistical analysis* - The results were expressed as the mean ± standard error of the mean (SEM) and statistically analysed using GraphPad Prism 8.0 (GraphPad Software, Inc., San Diego, CA, USA). A one-way analysis of variance (ANOVA), followed by Bonferroni’s test for multiple comparisons, was used to evaluate the lytic activity of anti-PL antibodies in the sera of *T. cruzi*-infected mice. For the detection of anti-PL antibodies and recombinant anti-PLA_1_ antibodies, a two-way ANOVA was performed, followed by Bonferroni’s multiple comparison tests.

## RESULTS

*Detection of anti-PL antibodies during experimental murine T. cruzi infection* - During the course of *T. cruzi* life cycle in the mammal host, the release of intracellular parasites from host cells could promote the exposition of lipid antigens and thus stimulate the generation of anti-PL antibodies. Therefore, we aimed to evaluate the presence of antibodies against PL (PE and PC) and their corresponding lyso-PL (LPE and LPC) in sera from mice infected with two different *T. cruzi* strains (RA or K98) by ELISA.

**Fig. 1:**
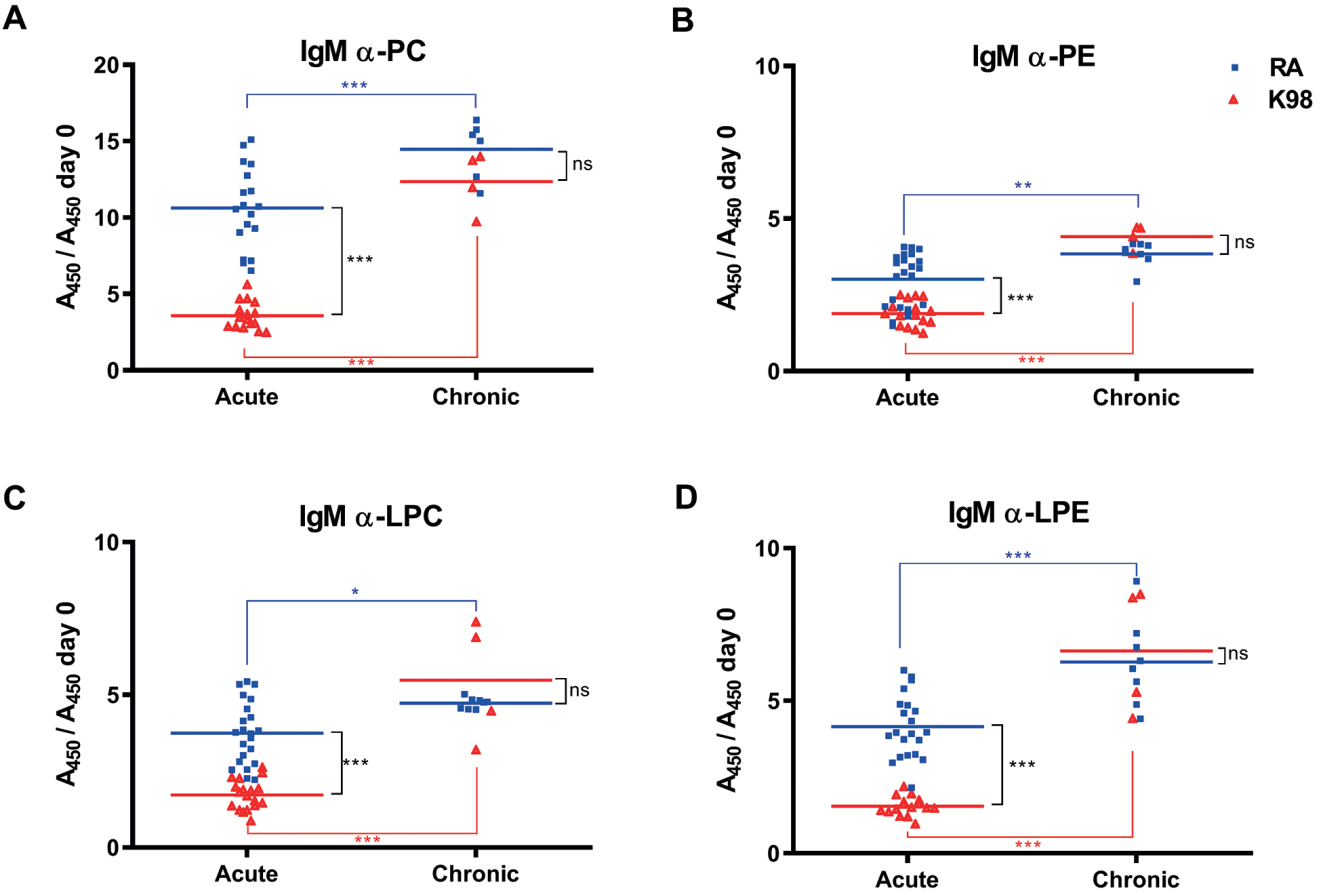
detection of IgM anti-phospholipid (anti-PL) antibodies in *Trypanosoma cruzi* infected mice. Detection of IgM antibodies against phospholipids: (A) phosphatidylcholine (PC) and (B) phosphatidylethanolamine (PE) and their corresponding lysophospholipids: (C) lysophosphatidylcholine (LPC) and (D) lysophosphatidylethanolamine (LPE), was carried out by enzyme-linked immunosorbent assay (ELISA). The results were expressed as the ratio between the absorbance at 450 nm (A450) of the serum of each infected mouse in relation to day zero (A450 day 0), uninfected mice. Each serum sample was tested in duplicate and the mean values for each group (RA or K98-*T. cruzi* infected mice) are indicated by horizontal lines. This image is representative of three independent experiments. Acute: sera from mice infected with RA or K98 *T. cruzi* strains from 21 to 54 days post-infection (dpi); chronic: sera from mice infected with RA or K98 *T. cruzi* strains at 120 dpi; blue square: mice infected with RA strain; red triangle: mice infected with K98 strain; ns: not significant; statistically significant: ^*^p ˂ 0.05, ^**^p ˂ 0.01, ^***^p ˂ 0.001.

As shown in Fig. 1, results indicate that mice infected with both *T. cruzi* strains generated significant levels of IgM anti-PL antibodies during the whole course of infection. The highest levels of IgM antibodies detected were against PC, being significantly higher in mice infected with the RA strain with respect to K98 during the acute phase (Fig. 1A). In the case of the other PL here studied, although the levels of anti-PE, anti-LPC and anti-LPE IgM were similar during the acute stage, animals infected with the RA strain showed higher IgM antibody levels compared to K98 (Fig. 1B, C and D). The results obtained in the chronic phase showed that all anti-PL antibodies increased and no differences were detected in the IgM antibody response developed by mice infected with both parasite strains.

**Fig. 2:**
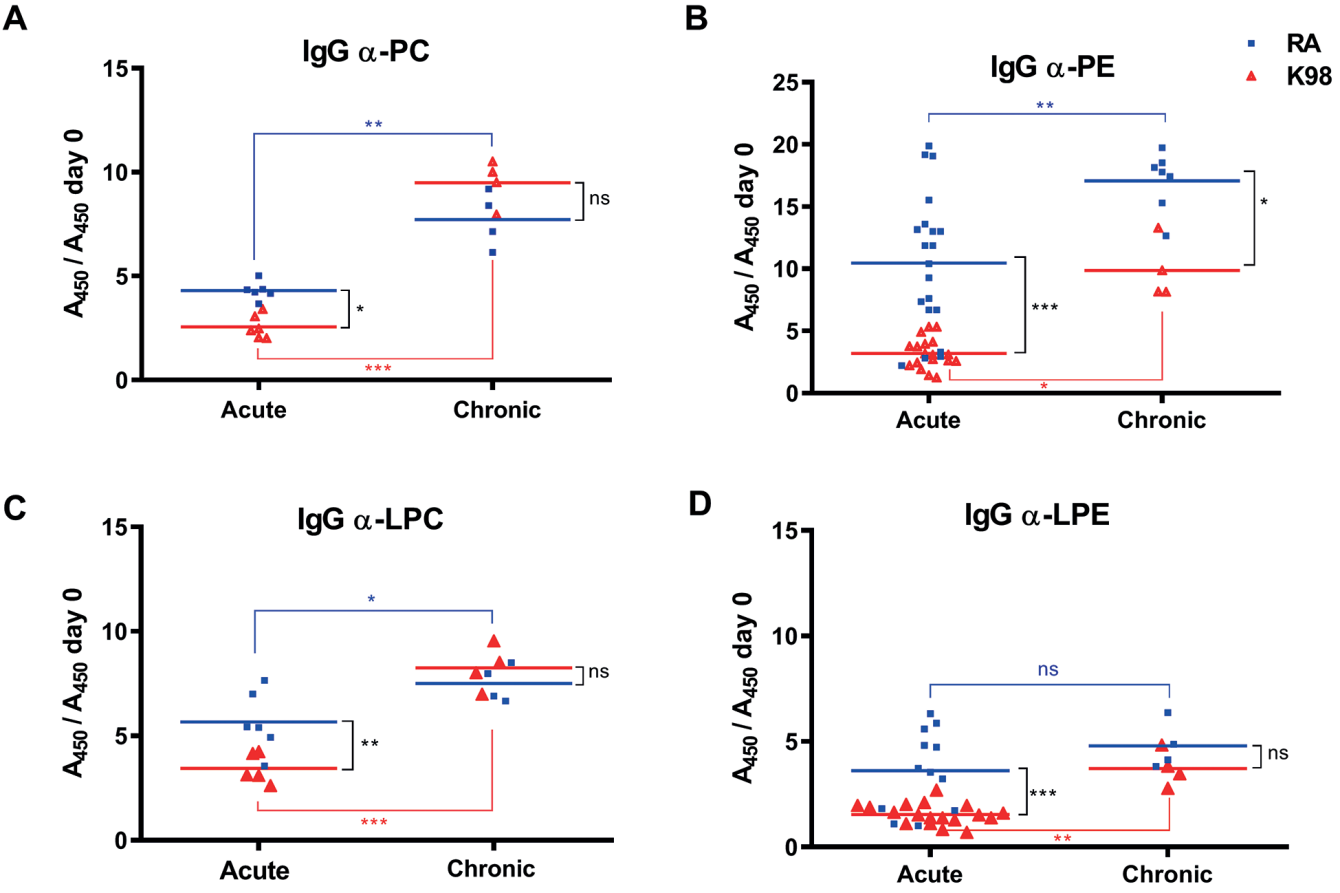
detection of IgG anti-phospholipid (anti-PL) antibodies in *Trpanosoma cruzi* infected mice. Determination of IgG antibodies against phospholipids: (A) phosphatidylcholine (PC) and (B) phosphatidylethanolamine (PE) and their corresponding lysophospholipids: (C) lysophosphatidylcholine (LPC) and (D) lysophosphatidylethanolamine (LPE), was carried out by enzyme-linked immunosorbent assay (ELISA). The results were expressed as the ratio between the absorbance at 450 nm (A450) of the serum of each infected mouse in relation to day zero (A450 day 0), uninfected mice. Each serum sample was tested duplicate and the mean values for each group (RA or K98- *T. cruzi* infected mice) indicated by horizontal lines. This image is representative of three independent experiments. Acute: sera from mice infected with RA or K98 *T. cruzi* strains from 21 to 54 days post-infection (dpi); chronic: sera from mice infected with RA or K98 *T. cruzi* strains at 120 dpi; blue square: mice infected with RA strain; red triangle: mice infected with K98 strain; ns: not significant; statistically significant: ^*^p ˂ 0.05, ^**^p ˂ 0.01, ^***^p ˂ 0.001.

The analyses of the IgG anti-PL response showed that, independently of the strain used, *T. cruzi* infected mice were able to produce significant levels of these antibodies during the course of parasite infection (Fig. 2). Remarkably, the highest levels of IgG antibodies detected were against PE, being significantly higher in mice infected with the RA strain compared to K98 during both acute and chronic phases (Fig. 2B). For anti-PC and anti-LPC IgG, results showed that mice infected with RA strain produced higher antibody levels compared to K98 during the acute phase, whereas in the chronic phase, although the titres of these antibodies increased, no significant differences were observed between parasite strains (Fig. 2A, C). Although higher levels of IgG anti-LPE antibodies were detected in the sera of RA-infected mice in the acute phase, no significant changes in the levels of these antibodies were detected along the course of infection. No differences between parasite strains were detected in the chronic phase (Fig. 2D).

*Determination of the lytic activity of anti-PL antibodies present in sera of T. cruzi-infected mice* - After confirming the presence of anti-PL antibodies in the sera of *T. cruzi*-infected mice and recognising the critical role of anti-*T. cruzi* antibodies in controlling parasitaemia during the acute phase of CD, we next assessed whether the anti-PL antibodies exhibited lytic activity. To do this, sera from infected mice were pre-treated with PC/PE liposomes, incubated with complement-sensitive epimastigotes, and the number of surviving parasites was measured as an indirect indicator of lytic activity.

**Fig. 3:**
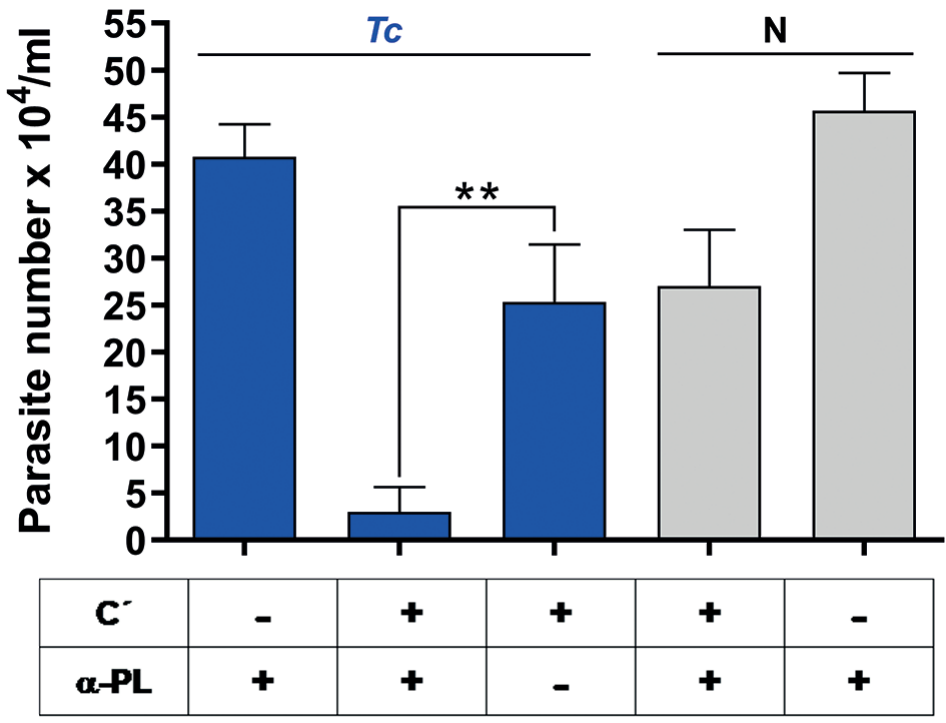
determination of the lytic activity of anti-phospholipid (anti-PL) antibodies present in sera of *Trypanosoma cruzi-*infected mice. *T. cruzi* epimastigotes (5x10^5^) were suspended in 250 μI of 1% bovine serum albumin (BSA)-phosphate-buffered saline (PBS) and incubated with 250 μI of dilutions of: inactivated sera from *T. cruzi*-infected mice (Tc, blue bars) or inactivated sera from non-infected mice (N, gray bars) or inactivated sera from *T. cruzi* infected mice that were pre-adsorbed with PC/PE liposomes to remove anti-PL antibodies (-αPL). The assays were performed in the presence or absence of an external source of complement (C´) (10 μI of non-immune guinea-pig sera), for 30 min at 28ºC. Aliquots were then taken, mixed with an equal volume of 4% paraformaldehyde to fix the parasites and these were counted in a Neubauer chamber by microscopy. Results represent the mean ± standard error of the mean (SEM) of triplicate determinations of two independent assays.

The results show a high degree of parasite lysis, measured as low parasite number, when exposed to sera from *T. cruzi*-infected mice with complement, without liposome pre-treatment, thus preserving anti-PL antibodies (Fig. 3, Tc +C’ +αPL). In contrast, sera from *T. cruzi*-infected mice pre-adsorbed with PC/PE liposomes (anti-PL antibodies removed) in the presence of complement (Tc +C’ -αPL), showed a moderate reduction in parasite lysis. This lytic effect was complement-dependent since parasites maintained their integrity when incubated with sera in the absence of complement (Tc -C’ +αPL). Additionally, comparing sera from infected mice containing anti-PL antibodies in presence of complement (Tc +C’ +αPL) to sera from non-infected mice under the same conditions (N +C’ +αPL), a significantly higher number of viable parasites were observed in the non-infected group. As a basal control, sera from non-infected mice in the presence or not of exogenous complement (N +C’ +αPL or N -C’ +αPL) revealed moderate lysis only in the presence of complement, suggesting the involvement of natural antibodies with lytic capacity.

Collectively, these results demonstrate that anti-PL antibodies generated during *T. cruzi* infection, display lytic activity against parasites in a complement-dependent mechanism.

**Fig. 4:**
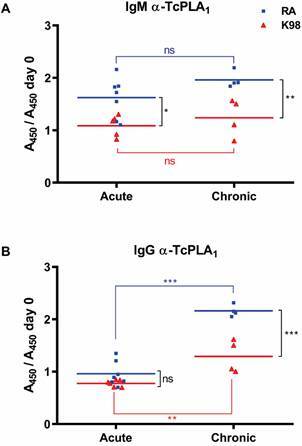
detection of anti-TcPLA_1_ antibodies in mice infected with *Trypanosoma cruzi* RA and K98 strains. Determination of anti-TcPLA_1_ antibodies was carried out by enzyme-linked immunosorbent assay (ELISA). The results are expressed as the ratio between the absorbance at 450 nm (A450) of the serum of each infected mouse in relation to day zero (A450 day 0). Each serum sample was tested duplicate and the mean values for each group are indicated by horizontal lines. This image is representative of three independent experiments. Acute: sera from mice infected with RA or K98 *T. cruzi* strains from 21 to 54 dpi; chronic: sera from mice infected with RA or K98 *T. cruzi* strains at 120 dpi; ns: not significant; statistically significant: ^**^p ˂ 0.01, ^***^p ˂ 0.001. TcPLA_1_: *Trypanosoma cruzi* Phospholipase A_1_.

*Mice infected with T. cruzi RA and K98 strains possess anti-TcPLA*_*1*_ *antibodies* - In previous work we determined that TcPLA_1_, an enzyme responsible for PL hydrolysis, is a virulence factor able to generate a significant humoral response in infected mice. Besides, antibodies against TcPLA_1_, generated during experimental murine infection, exhibited *in vitro* a neutralising effect and demonstrated the capacity to modulate PLA_1_ activity.^22^ Hence, in this work the presence of anti-TcPLA_1_ antibodies in sera of mice infected with RA or K98 *T. cruzi* strains was analysed by ELISA, using recombinant TcPLA_1_ as antigen. Fig. 4A shows that the levels of anti-TcPLA_1_ antibodies of the IgM isotype detected in the sera of RA-infected mice were significantly higher with respect to those in K98-infected mice, for both the acute and the chronic phase. When comparing the antibody levels within RA strain, no significant differences were observed between the acute and chronic phases. Similar results were obtained for K98-infected mice (Fig. 4A).

As concerns the IgG isotype, results indicate that during the acute phase no significant levels of anti-TcPLA_1_ antibodies were generated with respect to sera from mice at day 0. However, a significant increase in antibody levels was observed for both RA and K98 strains for the chronic phase with respect to the acute phase, in which sera from RA-infected mice presented higher anti-TcPLA_1_ antibodies levels with respect to K98 (Fig. 4B).

**Fig. 5:**
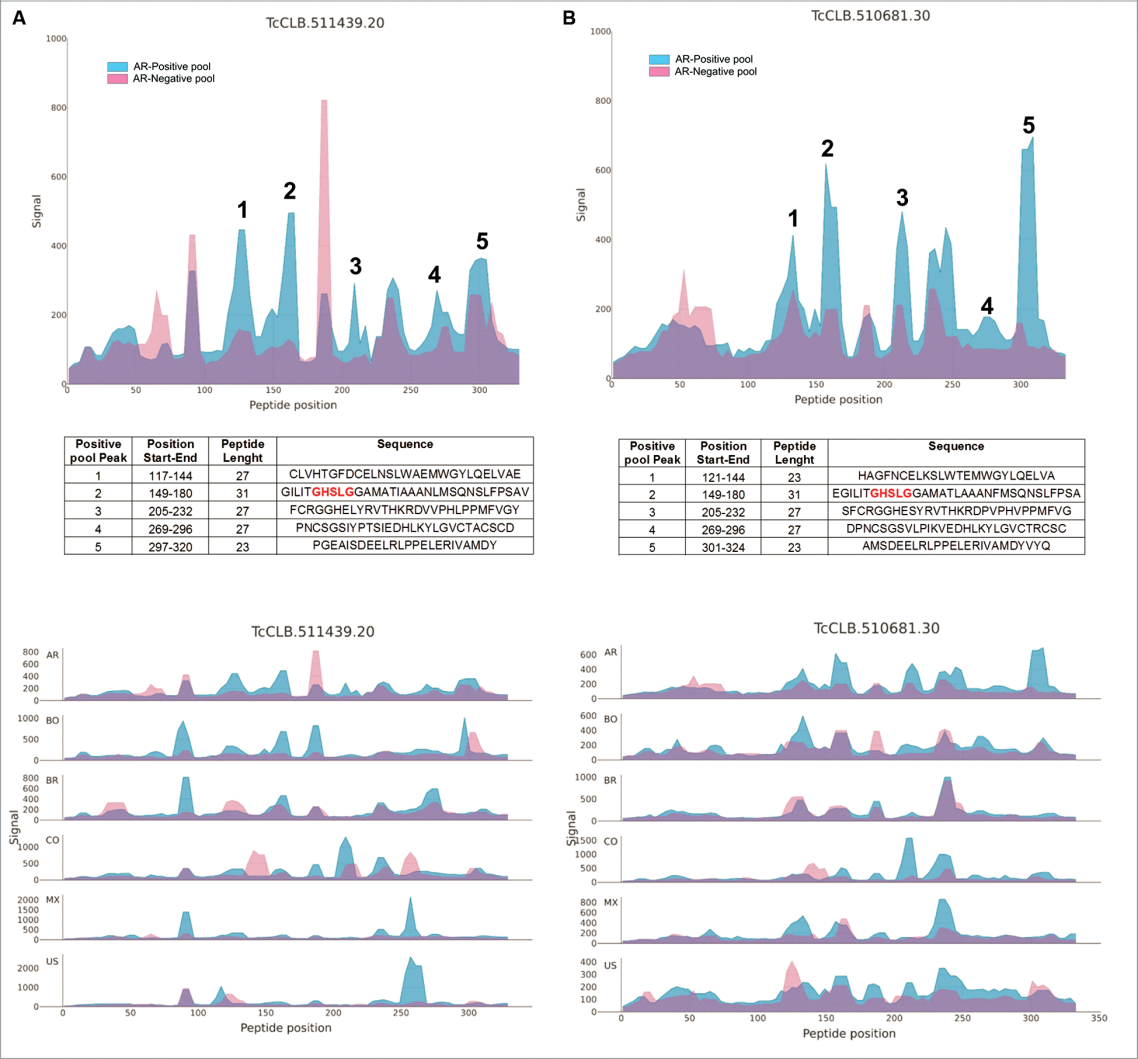
TcPLA_1_ antigenicity determination in Chagas-positive sample pools from patients of different geographic regions from the Americas. To identify the more reactive peptides of TcPLA_1_ (GenBank: AEX65839.1), the CHAGASTOPE-v2 resource was used. The *in silico* antigen search was performed using the corresponding homologous sequences of TcPLA_1_: TcCLB.511439.20 and TcCLB.510681.30 of the predicted proteomes of CLBrener31 strain. Results were displayed as graphs, which were constructed by the comparison of fluorescence signals for each peptide position of TcCLB.511439.20 (A) and TcCLB.510681.30 (B). The upper panels show the antibody-binding profiles, in blue for Chagas-positive sample pools obtained from Argentinian patients and in magenta for Chagas-negative pools from uninfected controls. Noteworthy, one of these antigenic regions includes the serine active site of the TcPLA_1_: GXSXG (in red). The lower panels show the antibody-binding profiles, in blue for Chagas-positive sample pools from different regions across the Americas: Argentina (AR), Bolivia (BO), Brazil (BR), Colombia (CO), Mexico (MX) and United States of America (US), and in magenta for Chagas-negative pools from uninfected controls. The y-axis shows arbitrary fluorescence units, and the x-axis shows peptide positions along the protein sequence chosen.

*TcPLA*_*1*_ *antigenicity determination by immunoinformatics* - The recent publication of the *T. cruzi* Antigen and Epitope Atlas CHAGASTOPE,^28^ allowed us to perform a comprehensive analysis on the antigenic properties of TcPLA_1_ and determined the amino acid sequences that presented greater antigenic recognition by the sera of CD patients from Argentina, with respect to controls. As shown in Fig. 5, five peaks with greater antigenic recognition by the Chagas-positive pool of human sera (in blue) were identified and correspond to peptide sequences ranging from 23 to 31 amino acids in length, each potentially representing distinct linear epitopes of antigenic regions within the TcPLA_1_ homologous proteins TcCLB.511439.20 and TcCLB.510681.30 (Fig. 5A-B, respectively, upper panels). Moreover, the proximity between certain peptide sequences implies the possibility of a contiguous larger antigenic region encompassing them.

In addition, the sera of CD patients from other geographical regions across the Americas, that comprise Bolivia, Brazil, Colombia, Mexico and United States of America, also recognised antigenic regions within the TcPLA_1_ homologous proteins (Fig. 5, lower panels) with different profiles.

## DISCUSSION

Parasite persistence can be a potent immune stimulus, leading to tissue damage and inflammation. *T. cruzi* molecules can mimic host proteins and induce autoimmune responses characterised by the production of autoantibodies and the activation of autoreactive cells. Despite the controversial nature of the involvement of autoimmunity in the progression of CD, evidence supports its role; however, the specific mechanism driving autoimmune responses in the progression of this disease remains unidentified.^29^ ^,^ ^30^ In recent years, some reports have indicated that anti-PL antibodies are elevated early in infections caused by protozoan parasites; however, whether they play a role in protection or contribute to pathology remains a subject of debate.^14^ ^,^ ^31^ ^,^ ^32^

In the present work, we demonstrate for the first time the presence of IgM and IgG isotype antibodies directed against PC and PE, the major cellular PL, as well as LPC and LPE (derived lysophospholipids), in the sera of mice infected with two *T. cruzi* strains belonging to different DTUs, during the acute and chronic phases of infection, with high titres persisting in the latter. This finding highlights the importance of PL as antigens during the course of experimental *T. cruzi* infection, as the only anti-PL antibodies detected to date have been against cardiolipin.^15^ In this sense, an association between positive serologic tests for CD and elevated levels of anti-cardiolipin antibodies has been demonstrated, but the clinical significance of this finding and the mechanism associated with the generation of anti-cardiolipin antibodies remains unknown. It has been suggested that cardiolipin may be one of the antigenic stimuli necessary for the development of Chagas heart disease, since it is found in significant amounts in the heart.^15^ In line with this finding, the high titres of anti-PL antibodies here obtained, which persist in the chronic phase of *T. cruzi* infection, led us to suggest their contribution to autoimmune phenomena, since elevated concentration of “non-classical” anti-PL antibodies, such as anti-PE antibodies, were detected in patients with anti-PL syndrome that presented thrombotic events or obstetric morbidity.^33^ Besides, other authors have reported elevated levels of anti-PC and anti-PE antibodies in paediatric patients diagnosed with cerebral infarction.^34^ In the case of protozoan infections, a study in patients with *Plasmodium falciparum* and *P. vivax* demonstrated that the production of higher levels of anti-phosphatidylinositol and anti-PC IgM antibodies positively correlated with malaria severity.^35^ Further reports have shown that anti-phosphatidylserine IgM and IgG antibodies titres are higher for *P. vivax* and *P. falciparum* and significantly lower in *P. knowlesi*, suggesting that these antibodies may contribute to anaemia in both *P. vivax* and *P. falciparum* malaria.^14^ ^,^ ^36^ In contrast, it has been described in *Leishmania major* that the presence of anti-PL antibodies enhance parasite phagocytosis by dendritic cells thus allowing a peculiar antigen presentation that leads to infection control.^37^

Herein, we demonstrated that pre-adsorption of anti-PL antibodies (using PC/PE liposomes) in sera from *T. cruzi-*infected mice resulted in a reduced complement-dependent lytic effect, suggesting that these antibodies may play a role in parasite control. Furthermore, during the acute phase of infection, the *T. cruzi* RA strain consistently induced elevated levels of antibodies (IgG and IgM) against all tested PL, likely due to its high replication rate and extensive cell destruction during this stage.^23^ In previous work, we demonstrated that *Babesia bovis*-infected cattle showed an increase in anti-PL IgM antibodies, with the attenuated R1A strain eliciting a greater anti-PC response than the virulent S2P strain, suggesting that these transient antibodies may partially limit the early phase of infection.^38^ Consistent with this, it has been reported that pre-immunisation of cattle with a lipid extract of *B. bovis*-infected erythrocytes resulted in reduced and delayed parasitaemia following challenge with virulent *B. bovis*.^39^ Similarly, the presence of anti-PC antibodies has been shown to reduce parasitaemia in *Plasmodium chabaudi chabaudi* challenge in mice.^40^ Furthermore, high titres of anti-phosphatidylinositol and anti-PC IgM are found in children with malaria, whereas those with cerebral malaria have significantly lower levels of anti-phosphatidylinositol IgM, suggesting that serum-derived anti-PL antibodies may contribute to anti-parasitic immune responses, by means of opsonisation and phagocytosis of parasitised erythrocytes and thrombocytopenia.^36^

On the other hand, reports on *Mycobacterium tuberculosis* infections describe that serum anti-PL antibody levels, specially IgM and total IgG, may serve as effective biomarkers for the diagnosis of pulmonary tuberculosis and monitoring the efficacy of anti-tuberculosis treatment, thus providing an alternative to standard methods, as anti-PL IgM levels significantly decreased after drug treatment.^41^ ^,^ ^42^

As regards antibodies against TcPLA_1_, the differential humoral responses against recombinant TcPLA_1_ detected in the sera from mice infected with RA or K98 strains, could be attributed to differences in their specific parasitaemia peaks, tissue tropism and DTUs.^2^ ^,^ ^23^ ^,^ ^24^ Moreover, the increasing levels of anti-PLA_1_ IgG antibodies in the chronic phase of infection for both strains suggests that these antibodies may contribute to host control of parasitaemia since we already demonstrated that the presence of anti-TcPLA_1_ antibodies in the sera of *T. cruzi* infected mice with RA, K98, and CvD strains, have shown to modulate TcPLA_1_ activity and inhibit the invasion of non-phagocytic cells.^22^ In this regard, the *in silico* analyses in the CHAGASTOPE resource demonstrated that the pool sera of CD patients recognised in TcCLB.511439.20 and TcCLB.510681.30, homologous proteins of TcPLA_1_, a linear epitope that possess the catalytic active site of the enzyme, thus supporting that anti-TcPLA_1_ antibodies might be able to inhibit/neutralise the enzyme activity. Further studies are needed to determine the extent to which anti-TcPLA_1_ antibodies contribute to the reduction of parasitaemia in order to evaluate the potential of this antigen as part of an immunoprophylactic strategy to control CD. In this context, and given the extensive genetic diversity of *T. cruzi* in diverse regions, expansion of *in silico* studies using sera from patients in different geographical areas will be essential to gain a more complete understanding of regional variations in immune response.

In the present work, we determined the presence of IgM and IgG antibodies against the major cell phospholipids and their derived lysophospholipids during experimental *T. cruzi* infection, as well as TcPLA_1_ antibodies, since PL hydrolysis is mainly due to this enzyme activity. In addition, the use of the CHAGASTOPE resource allowed us to predict whether TcPLA_1_ could induce an immune response in CD patients from different geographical regions. Our findings are expected to contribute to a better understanding of the presence and role of anti-PL and anti-TcPLA_1_ antibodies in infectious diseases such as CD. This knowledge may aid in the validation of new diagnostic markers, the monitoring of treatment response, and the development of potential therapeutic targets for diseases in which PL and PL-related enzymes are relevant antigens.
